# Dr. Rennet’s Rejoinder

**Published:** 1850-11

**Authors:** 


### Dr. Bennet's Rejoinder.

*On the frequency of Ulceration of the Cervix Uteri, and on the Pathological value of the term Ulceration.* By J. Henry Bennet, M. D., Late Physician-Accoucheur to the Western Dispensary, etc.

*To the Editor of The Lancet.*

Sir,—In the August No. of The Lancet there has appeared a paper of Dr. Tyler Smith, previously read at the Westminster Society, on the "Supposed Frequency of Ulceration of the Os and Cervix Uteri," to which I trust you will allow me briefly to reply.

I would firstly remark that the title of Dr. Smith's paper ought not to have been on the "presumed" frequency of ulceration of the cervix, but rather "On the Pathological meaning of the term Ulceration, as applied to solutions of continuity of the organ in question." The, in reality, erroneous title of Dr. Smith's paper implies a negation, on his part, of the frequency of the lesions which I have described in my work on Inflammation of the Uterine Organs, under the generic head of Ulceration; whereas such a negation is not to be found in any part of his paper. On the contrary, he admits, in a dozen places, the probable frequency of these lesions, but differs from me in thinking that the term ulceration ought, in sound pathology, to be applied to them.

This is a most important feature in the discussion raised by Dr. Smith; inasmuch as by thus correcting the title of his paper, and making it really agree with the contents of the latter, we reduce the question at issue to what it actually is, to one of *words only*—viz., are the lesions I describe in my work to be considered forms of ulceration, or are they not ?

Before I proceed to the examination of this question, I must observe, that although evidently written with a very different purpose, Dr. Tyler Smith's Essay, from first to last, substantiates and supports my views. By a process of mental reasoning and reflection, if not by clinical experience, he has clearly become a convert to my doctrines respecting uterine pathology and so fully appreciates the influence of the various causes of inflammatory disease to which the uterine organs are exposed, at the various periods of uterine life—causes which I have developed at length in my work, and which he rapidly enumerates—that he absolutely states, "*If we consider excoriation or abrasion as genuine ulceration, probably no woman ever passes through life without suffering from this form of disease.*" Surely this is out-heroding Herod. Although my *clinical* experience has enabled me to say that out of 300 women presenting decided uterine symptoms, and observed in the course of two years' special practice at a large public institution, 222 offered some form or other of ulceration, (*including* abrasions and excoriations,) I should be very sorry indeed to make such a sweeping assertion as the above; uterine lesions even of this description, not existing long, without making known their presence by local or constitutional disturbance at least, in the immense majority of cases. Were this assertion true, we then should find nearly every other female we meet laboring under uterine disease, as, I hear, some practitioners facetiously state to be the case at present. I leave Dr. Tyler Smith, however, to reconcile his theory of what ought to be the state of uterine pathology with what it is clinically found to be, and will only add, that, for my part, although convinced of the frequency of inflammation, and of inflammatory ulcerative lesions of the cervix in women presenting uterine symptoms, I still have great confidence in the power of nature to carry through her operations, generally speaking, without hurt or accident. I consequently decidedly think that the very great majority of women pass through all the trials of uterine life without being affected either with abrasions or excoriations. Inflammatory lesions are no more *the rule* with the uterus than with the brain, the liver, or the lungs, although they may be as frequently, or even more frequently, the exception.

With regard to the main question at issue, the proper interpretation of the term ulceration, in applying it generally to all solutions of continuity of the cervix and its cavity, whether slight or considerable, I have followed both the highest authority and the bent of my own mind. I have always considered divisions and distinctions in the nomenclature of disease as useless, and obstructive to progress, unless founded on some real and therapeutically important difference. Thus, were I to write a treatise on diseases of the skin, I should throw aside, without scruple, one-half of the forms of disease now admitted, because they are merely founded on a species of botanical consideration of the visible appearance of the disease, and are, in reality, only different modes of manifestation, and different stages of development, of a malady identically the same, and requiring the same treatment.

Applying these principles of general pathology to the ulcerative lesions produced by inflammation of the cervix and its cavity, instead of describing abrasions, excoriations, and luxuriant ulcerations, as distinct morbid conditions, (which they are not,) I have embodied them all in the general term ulceration, adding, by way of explanation, "that ulceration occupying the cervix uteri may present all the various modifications which suppurating surfaces offer in any other part of the body, *from the minute granulation of a slight abrasion to the livid vegetations of an unhealthy sore.*"

That I am warranted in applying the term ulceration even to a mere abrasion, the result, not of physical violence, but of inflammation and of morbid vital action, must be evident to all who are acquainted with the classical literature of the profession. "Ulceration," says Samuel Cooper, "is the process by which *sores* or ulcers are produced in animal bodies." J. L. Petit defines an ulceration, or ulcer, "as a solution of continuity, from which is secreted pus, or a puriform, sanious, or other matter." Boyer states, that "an ulceration is a solution of continuity of the soft parts, more or less ancient, accompanied by a purulent secretion, and kept up by some local or internal cause." Any of these definitions apply quite as truly to a mere abrasion or excoriation, secreting pus or sanies, as to the chronic excavated *cutaneous* ulcer, which Dr. Tyler Smith most unaccountably expects to find on the cervix uteri.

Indeed, it is with extreme surprise that I find Dr. Tyler Smith saying, that, to be able to apply the term ulceration to the cervix uteri, "we must look for a solution of continuity with a secreting surface, separated from the healthy structures, having defined edges, everted or inverted,—for an ulcer, in fact, (query, a cutaneous ulcer?) in the common pathological meaning of the term." Had not Dr. Smith commenced by stating that he is in the habit of using the speculum, I should be inclined to think that he was reasoning from analogy only; for the fact is, and he ought to know it, if his opinions are the result of practical experience, that the form of ulcers thus described is scarcely ever met with on the neck of the uterus, except as the result of syphilitic chancre or of corroding ulcer. Owing to the tenuity of the mucous membrane lining the cervix, and its cavity, the margin of an inflammatory ulceration is scarcely ever, if ever, either everted or inverted. So much is this the case, that it is generally most difficult to say where the ulceration finishes, until, by the application of the nitrate of silver, the margin of the sore, or the point where the epithelium finishes, be revealed. In my work (see page 103) I have distinctly stated, and now repeat, that "whatever the character of an inflammatory ulceration of the cervix, the ulcerated surface is *never* excoriated; it is always on a level with or above the non-ulcerated tissues that limit it, and its margin never presents any abrupt induration. Owing to this circumstance it is always impossible to determine by the touch the precise point at which the ulceration terminates."

Thus Dr. Tyler Smith's criticism of my position respecting the frequency of uterine ulceration, is founded, on the one hand, on a frivolous negation of the term ulceration to abrasions and excoriations, these lesions being strictly and legitimately within the pale of all classical definitions of ulceration; and, on the other, on the establishment of a visionary ideal of uterine ulceration, drawn from chronic cutaneous ulcers of the leg, such sores o "ulcers" not being met with at all, in reality, on the cervix uteri.

In the course of the above argumentation, I have not alluded to the French writers on uterine diseases, because I did not require their assistance to establish my position. I may now, however, add, that all who have written in France on the subject of uterine inflammation, have applied the word ulceration to the lesions to which I apply it. Some have called abrasions and excoriations, exulcerations, or granular ulcerations, which I have not thought proper to do; but this is the only difference between me and them. I have thus not only reason and pathology on my side, but also antecedents, and the example of my seniors—men of the first eminence and talent, such as Lisfranc, Gendrin, Jobert, Duparcque, &c.

Dr. Tyler Smith, in common with all those who are now *vainly* endeavoring to arrest the strong current of professional feeling towards a correct and sound uterine pathology, appears to think that I and those who adopt my views are discarding all constitutional treatment, and directing our attention solely to local lesions. This, however, is a most unwarranted "petitio principis." I would ask—Where is the proof? Certainly not in my work on Uterine Inflammation, for a large portion of it is devoted to the minute and careful investigation and elucidation of general and constitutional symptoms, to which I strenuously and continually direct the attention of my readers. Nor have I observed any evidence, in the practice of those medical men, converts to the modern doctrines, with whom I come in contact, that constitutional means of treatment are by them neglected.

Dr. Tyler Smith lays great stress on the results of the post-mortem examination, at St. George's Hospital, which seem to me, on the contrary, to completely corroborate my views. He states, that only seven instances of ulceration of the cervix (excavated with distinct edges, I suppose) have been found on the post-mortem examination of the females who die annually in the hospital. In the same paragraph, however, he is obliged to confess that slight abrasions, discolorations, and granulations, were frequently observed. The severe ulcerations alluded to appear to have been scrofulous sores, the result of the softening and emptying of the tubercular masses, or malignant ulcerations.

I cannot conclude these remarks, without entering my protest, in the most forcible and energetic manner, against a statement made by Dr. Tyler Smith, at the conclusion of his paper. He says:—"At the present time, a veritable uterine panic affects the upper and middle classes of society, and every woman with the slightest ache or discharge, is not satisfied until the peccant organ has been ocularly inspected." I have no hesitation whatever in stating that this assertion is a libel on our country-women, which I trust has only escaped from Dr. Smith in the hurry of composition. The change that has taken place in the arguments brought forward by those who adhere to the prejudices and errors of days which we shall soon be able, I firmly believe, to call past, is truly remarkable. Five years ago, when I published the first edition of my work, I was greeted by the observation, that English women were too modest and reserved to submit to physical examination, and that I should only destroy my professional prospects by advancing views which required it. Now, however, that English women suffering under uterine disease are beginning to become aware that there is relief to be found, and that they need no longer be handed from practitioner to practitioner, in a vain search after health; now that they show themselves willing to control agony of mind and pain of body, for the sake often of those who are dear to them, an odious accusation is thrown in their teeth, and they are told that they are ready, nay, *anxious*, to submit to uterine examinations, nearly without a cause or pretext. I can only say, that I meet with *no such females*, either in the higher or the lower ranks of my country-women, and that I blush for those who thus asperse them.

I am, Sir, your obedient servant,

HENRY BENNET.

